# Durability of PS-Polyurethane Dedicated for Composite Strengthening Applications in Masonry and Concrete Structures

**DOI:** 10.3390/polym12122830

**Published:** 2020-11-28

**Authors:** Konrad Kwiecień, Arkadiusz Kwiecień, Teresa Stryszewska, Magdalena Szumera, Marta Dudek

**Affiliations:** 1 Faculty of Materials Science and Ceramics, AGH University of Science and Technology, 30-059 Krakow, Poland; mszumera@agh.edu.pl; 2 Faculty of Civil Engineering, Cracow University of Technology, 31-155 Krakow, Poland; akwiecie@pk.edu.pl (A.K.); tstryszewska@pk.edu.pl (T.S.); marta.dudek@pk.edu.pl (M.D.)

**Keywords:** polyurethane, flexible-joints, durability, degradation, thermal analysis

## Abstract

Polyurethane flexible joints (PUFJ) and fiber reinforced polyurethanes (FRPU) have shown great potential in the repair and protection of masonry and concrete structures. However, some questions have been raised about the durability of such solutions. The accelerated weathering and thermal stability tests carried out so far have shown the mechanical stability of PS-polyurethane in temperatures up to 100 °C and some UV-induced surface degradation. The paper reports the results from tensile tests of PS-polyurethane, used in the technologies mentioned above after being subjected to aging in different corrosive factors, a thermal analysis of unaged polymer which consists of DSC-TGA and dilatometry studies, and SEM-microscopy observation of the specimens with the indication of the elemental composition (EDS). PS-polyurethane showed low sensitivity to weathering with exposition to UV-radiation, some reactiveness to aqueous environments of a different chemical nature, and resistivity to soil and freezing in both air and water. SEM observations indicated changes in the composition of mineral fillers as the main effect of immersion in different water solutions. DSC-TGA studies showed the thermal stability of PS-polyurethane up to 200 °C and degradation proceeding in five stages. Dilatometry studies revealed that the first-degree thermal degradation over 200 °C causes a serious loss of mechanical properties.

## 1. Introduction

Although technology has grown quickly in recent years, natural disasters still remain a serious problem worldwide, generating unexpected losses of both lives and property. According to UNICEF, there were more than 60 mln people affected by climate-related and geophysical events in 2018 [1]. One of the major cause of deaths in case of an earthquake are failures of buildings and other masonry structures due to their brittle behavior. It was proven that such failures can be successfully avoided or at least delayed in time with different technologies based on polyurethanes (PU) and their fibrous composites [2,3,4,5,6,7]. Polyurethane flexible joints (PUFJ) and fiber reinforced polyurethanes (FRPU) have been introduced in the last decade and investigated in laboratory and in-situ tests concerning their mechanical properties. PUFJ and FRPU were tested as structural bonding systems, which transfer high loads and high deformations simultaneously. This ability was utilized in the repair of cracked masonry and concrete structures using PUFJ [2,5,6,7]. They have been working in various environmental conditions and under the influence of chemical agents [7]. Various types of PU mass have been investigated in PUFJ, and PS-polyurethane—polymer of PS-type (Sika PS)—has been used in the PUFJ technology for fast bonding of prefabricated PU laminates to concrete RC frames [8,9]. Ductile properties of PU materials and their damping ability have been tested in steel and aluminum structures as adhesive bonding layers [10,11]. The ability of flexible PU adhesives to redistribute shear stress over a large area, unlike stiff epoxy [12] and mineral adhesives [13], caused the development of these adhesives in the composite strengthening of masonry and concrete structures. PS-polyurethane has been applied as a flexible adhesive layer and matrix of composite fibers in FRPU systems, bonding composite laminates to concrete elements as externally bonded (EB) systems [4,14] and near-surface mounted (NSM) systems [14]. PU flexible adhesives and matrices were also used as FRPU in masonry applications [3,15,16]. However, there have been concerns about the durability of the provided solutions, as they were all tested shortly after implementation. Materials used for crack reparation and masonry structures protection have to be able to withstand different chemical compounds, humidity, heat, cold, and other corrosive factors present in the place of application, and therefore this work focuses on preliminary research on the resistance of polymer products used for the mentioned technologies to the most usual corrosive environments.

**Figure 1:**
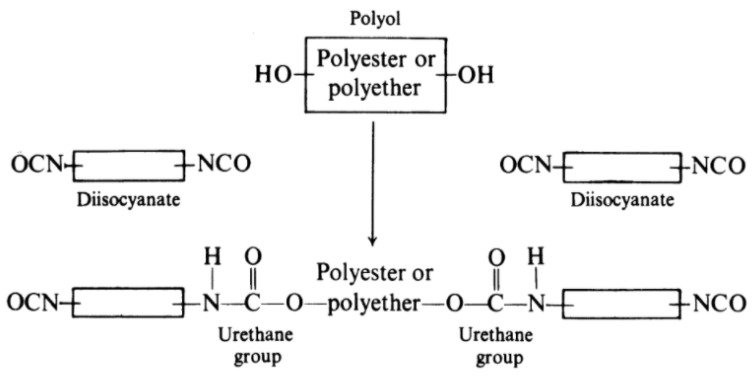
Typical synthesis reaction of polyurethanes [18].

Polyurethanes are a large group of polymers which contain urethane links in their structure. There are plenty of possible paths to obtain urethanes [17], the most common substrates however are different isocyanates, polyols, and chain extenders (Figure 1). Each of the substrates also include other chemical groups that, combined with individual synthesis paths, lead to a wide range of mechanical, chemical, and physical properties [18].

**Figure 2:**
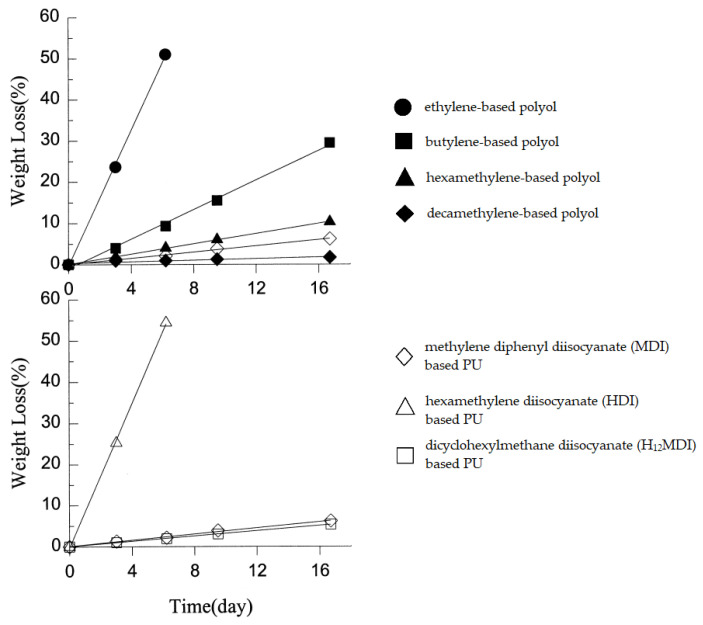
Influence of different substrates used for PUs synthesis on degradation rate in 10% NaOH solution at 37 °C. Upper graph: PUs obtained from polyols with different aliphatic segments. Lower graph: PUs obtained from different diisocyanates [23].

Thanks to polyurethanes’ versatility, they are applicable in various fields. Unfortunately, many degradation factors are affecting their properties. In general, there are several degradation types of polymers: photo-oxidative, thermal, ozone-induced, mechanochemical—chemical changes caused by mechanic factors e.g., ultrasounds, chemical by organic or inorganic compounds, catalytic, hydrolytic, and biodegradation [19,20]. In masonry structures for civil engineering, the main environmental degradation factors are alkalis, as mortar in a large degree consists of water and calcium hydroxide. Polyurethane foam waste was investigated with promising results as an alternative aggregate for lightweight concrete which implies its compatibility with mortar [21]. A more detailed study of this topic showed however that the PU-aggregates are likely to react with strong alkalis (NaOH) [22]. PUs’ resistance to NaOH was shown to be highly dependent on the length of aliphatic chains and molecule flexibility of isocyanates (Figure 2). Polymers obtained from substrates with longer hydrophobic chains showed much less weight loss. In turn, flexible, linear isocyanate PU degraded much faster than stiffer, cyclic isocyanate PUs [23]. Urethane link itself is resistant to hydrolysis, but some PUs may not be due to other chemical groups present in their structure [20], what is especially visible in contact with seawater—some PUs show significant weight loss after 12 months incubation, while others seem to have hardly any changes in their structure within this time [24].

The other corrosive factor for PU-based flexible joints is UV radiation if technology faces outdoor exposure. This kind of degradation affects mostly the polymer’s surface, as Atomic Force Microscopy (AFM) studies and coating roughness measurements showed significant changes in polymer’s surface topography [25]. This is because there is a limited penetration depth for UV radiation. In practical applications material faces outdoor weathering—a combination of photo- and thermal oxidation due to different electromagnetic wavelengths of solar origin [26]. The main mechanism of thermal oxidation is, similarly to photooxidation, the formation of free radicals and further reactions. It appears however in bulk what is much more affecting mechanical properties of the material [26]. In normal (environmental) conditions, polymer within masonry structures faces relatively low heat (except facades exposed to sun radiation, e.g., in desert areas) for an elongated time. Thermal oxidation may be most dangerous in case of fire when the temperature rises to several hundred degrees. Termogravimetry (TGA) studies of different PUs show that these polymers usually do not disintegrate below 200 °C, and the first stage of degradation is most often urethane link decomposition [20,27,28,29,30]. Biodegradation is another possible path of PUs’ degradation, as they may have contact with microorganisms in water or soil in the place of application. Among the microorganisms commonly found in nature, fungi seem to be the most dangerous for PUs, as some of their enzymes can decompose the urethane bond [31,32].

The durability of the mentioned polymers was tested so far in accelerated weathering [33] showing that the UV-induced degradation takes place on polymers surface, affecting the mechanical properties of the material when thin layers are applied. The authors suggest using a protecting coating for this technology if possible [33]. The polymers were also investigated as FRP composites for bricks reparation in the field of thermal aging and salt decay [34]. It was shown that heating cycles may cause delamination from brick when stiff epoxy resins are applied (not in the case of polyurethane adhesives), or salt crystallization may occur, although the problem concerns the composite-brick boundary, not the polyurethanes itself [34]. The influence of different corrosive factors was tested on the other type of composites—steel-reinforced polyurethanes—too. Again, any failure noticed was about metal reinforcement, rather than polymer matrix [35]. This all generates the question of how durable the mentioned PUs themselves are, especially, for technologies based on sheer polymers—PUFJ. Thermal stability of mechanical properties of PS-polyurethanes was previously tested but only up to 100 °C, showing no significant effect of temperature changes within the range 20–80 °C on Young’s modulus in pure polymer or strain distribution in CFRP composites [36,37].

This paper presents an evaluation of PS-polyurethane specimens, prepared for tensile tests according to ISO 527 standard [38]. The specimens were subjected to 1000 h aging in different corrosive environments appearing in the places of application: heat, cold, distilled water, seawater, alkali solution, atmospheric conditions, and soil. After that tensile tests were executed. Destruction areas of specimens were investigated by scanning electron microscopy (SEM) and energy dispersive spectroscopy (EDS) technique. Moreover, thermogravimetry (TGA), differential scanning calorimetry (DSC), and dilatometry tests were provided to obtain more detailed information on the heat resistance of the pure polymer. 

## 2. Experimental Setup

### 2.1. Materials and Sample Formation

Polyurethanes that are subject of the study were of PS-type (Sika PS)—the new product in the testing phase. After 7 days of cross-linking at room temperature, the samples were excised according to ISO 527 standard [38].

### 2.2. Degradation Studies

Specimens prepared as in Section 2.1 were subjected to the different corrosive environments for 1000 h.

- Reference: air 20 °C (room conditions)
- Weathering: atmospheric conditions (16–31 °C, up to 5 mm/day precipitation), unshaded, uncovered
- Soil: buried 5 cm deep, atmospheric conditions (16–31 °C, up to 5 mm precipitation), unshaded, uncovered
- Distilled water: 60 °C
- Alkalis at room temperature: water solution Ca(OH)_2_ 2 g/L, 20 °C
- Alkalis hot: water solution Ca(OH)_2_ 2 g/L, 60 °C
- Seawater: 20 °C,
- Tap water: 20 °C
- Frozen tap water: −16 °C
- Cold air: −16 °C

**Figure 3:**
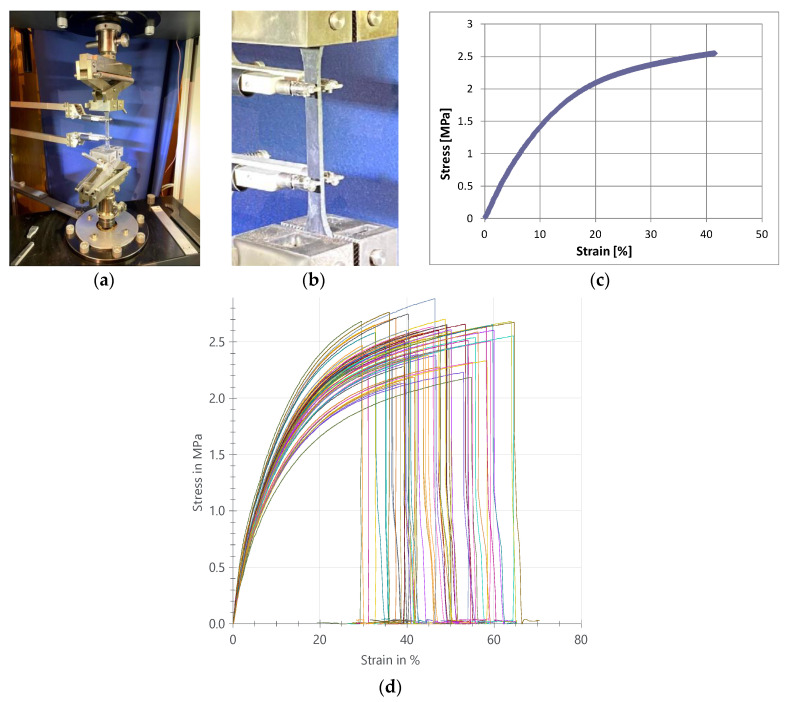
A sample prepared for the tensile test: (**a**) setup from a testing machine, (**b**) PS-polyurethane dog-bone specimen with long-travel extensometer, (**c**) non-linear stress-strain characteristic of a reference (I) specimen in the tensile test, (**d**) stress-strain characteristics range of all specimens (I-X) tested in tensile.

After incubation, a tensile test [38] was performed on all samples (6 samples per each environment) by testing machine ZWICK 1455 20 kN with scissor clamping of 10 kN (Figure 3a). Standard dog-bone specimens [38] were prepared and pre-force 0.01 MPa, tensile modulus displacement ratio 50 mm/min, and test displacement ratio 50 mm/min (strain ratio 100%/min) were applied to all tested polyurethane specimens (aged and reference). A long-travel extensometer 600 mm with the accuracy of Class 1 and a resolution of 1 μm was used for a precise measure of strains (Figure 3b). In all cases, stress-strain characteristics manifested a similar non-linear character to the reference tested specimen (Figure 3c). Derived results in terms of graphs are presented in Figure 3d for all 60 tested specimens, showing the variation of obtained curves with ultimate stress (2.2–2.9 MPa) and ultimate strain (30–65%). All specimens manifested the same failure mode—break in the measuring range of the extensometer.

Evaluation of the obtained results included a comparison of statistical significance calculated via Tukey test [39] in software Origin 2020b of four parameters obtained from the tensile curve (Figure 3c): initial Young’s modulus (*E*_0_), tensile strength (σ_M_), maximal strain (ε_M_), maximal work (*W*_M_), and one additional parameter: equivalent Young’s modulus (*E*’), fulfilling linear simplification according to the Hook’s law [40,41], which was calculated from linear regression of stress values related to strains at 1%, 2%, 3%, 4%, 5%, and 6% elongation as

σ=E′ϵ′

$$\;\epsilon^{\prime }=\frac{\ln\left(1+\epsilon \right)}{1+\epsilon }\;$$

where *E*’—equivalent Young’s modulus, ϵ′—equivalent strain, σ—nominal tensile stress, and ϵ—engineering strain.

### 2.3. Thermal Analysis

All of the thermal analyses were carried out on either grated (DSC-TGA) or excised (dilatometry) fragments of reference (I) specimens after tensile tests. DSC-TGA analysis was conducted in STA 449 F3 Jupiter 7 (NETZSCH-Gerätebau GmbH, 95100 Selb, Germany) operating in the heat flux DSC mode in the atmosphere of synthetic air (40 mL/min). The applied temperature range was between ambient to 600 °C with a rate of heating 10 °C/min. 6 samples were in the form of granules (6 mg) and were placed in aluminum crucibles. Dilatometric measurements were conducted in DIL 402 C dilatometer (NETZSCH-Gerätebau GmbH, 95100 Selb, Germany) in the atmosphere of synthetic air. The applied temperature was between ambient and 240 °C with a rate of heating 5 °C/min and the force range (load at the sample) was 10 mN. Three samples excised to length 9.8–10 mm were placed in a holder system made out of Al_2_O_3_. 

### 2.4. SEM Observatrions and EDS Evaluation

Observations of the microstructure of the samples were carried out by scanning microscope Carl Zeiss EVO-MA 10 using a BSD detector (Carl Zeiss Microscopy, Jena, Germany). At the same time, elemental composition analysis was performed using the EDS Bruker XFLASH 6/30 detector (Bruker, Hamburg, Germany). EDS analysis results were presented in qualitative (mapping) and quantitative (table) form. Because of the conductive nature of the tested materials, observations were made in a high vacuum on non-dusted samples, with the accelerating voltage EHT equal to 20 kV. The observations were performed on the surface of breaking-up. Additionally, microanalysis under a microscope was also conducted for crystals that formed on the polymer surface of specimens subjected to distilled water at 60 °C (IV). 

## 3. Test Results 

### 3.1. Degradation Studies

**Figure 4:**
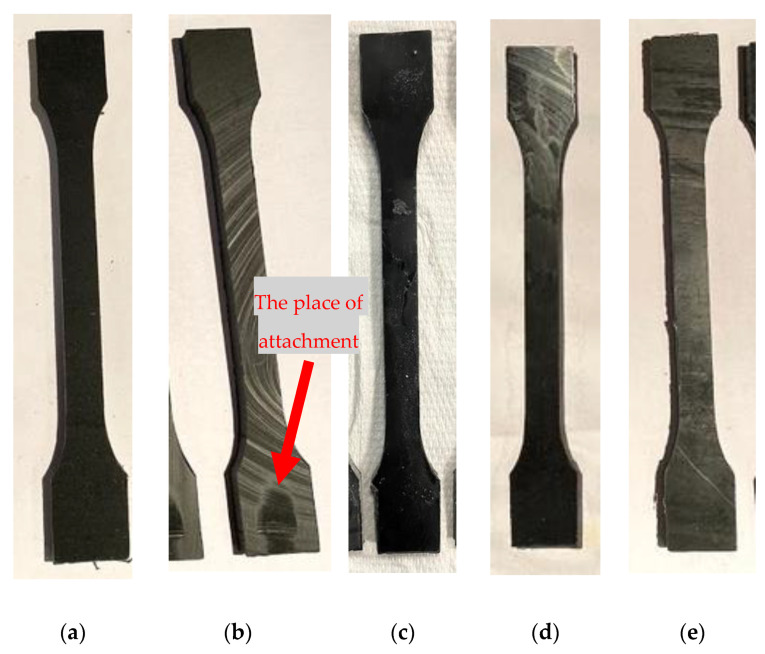
Representative samples with some visible effects on the surface compared to the unaged one. (**a**) reference (I), (**b**) weathering (II)—red arrow shows the place of attachment, which was covered from the sun radiation, (**c**) alkalis at 20 °C (V), (**d**) tap water (VIII), (**e**) frozen water (IX).

**Figure 5:**
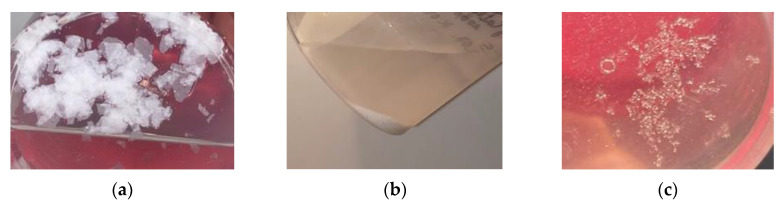
Solid residue in immersion fluids (**a**) Alkalis at 20 °C—white crystals in the shape of petals, (**b**) Alkalis at 60 °C—white suspension of fine particles, (**c**) white/colorless fine crystals.

After removing the samples from corrosive environments, no structural changes were observed, although certain delicate effects were visible on the surface of some samples (Figure 4). The obtained patterns consisted of a residue similar to dust that was removable by abrasion. In three cases of immersion fluid (distilled water (IV) and alkalis both temperatures (V, VI)) solid residue of different morphology was noticed (Figure 5). In alkali solutions, the precipitate was white and in the shape of petals in lower temperature and fine powder in higher temperature. The residue should have the same chemical nature, but the different form might be an effect of crystallization kinetics influenced by temperature. In distilled water the precipitate was in the form of small transparent and colorless crystals, what indicates ionic substances elution from the material. 

**Figure 6:**
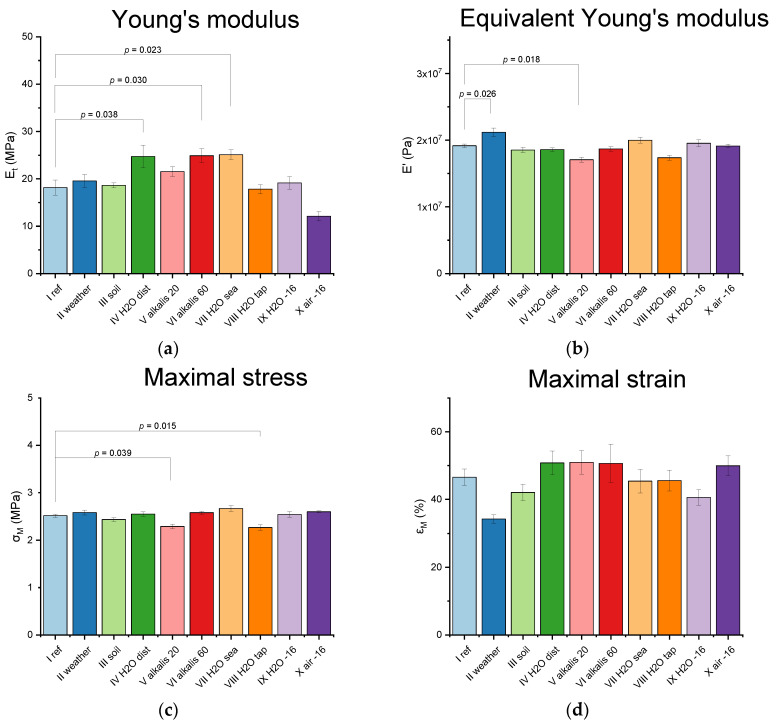

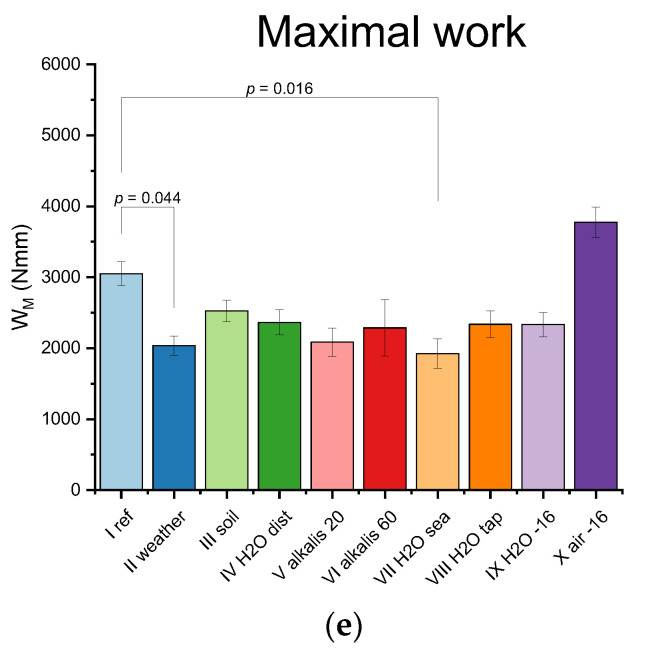
Results of Tukey test comparing mechanical properties—(**a**) Young’s modulus, (**b**) equivalent Young’s modulus, (**c**) maximal stress, (**d**) maximal strain, and (**e**) maximal work—of aged samples with references (cases with the recognized impact of corrosive factors marked with *p*-values).

For provided tensile tests mean values and standard derivations were calculated and compared via Tukey test for initial Young’s modulus—*E*_0_ (Figure 6a), equivalent Young’s modulus—*E*’ (Figure 6b), tensile strength (maximal stress)—σ_M_ (Figure 6c), maximal strain—ε_M_ (Figure 6d), maximal work (area under the curve in Figure 3c)—*W*_M_ (Figure 6e). The Tukey test showed if there were statistically significant differences. Three significance levels were applied: *p* < 0.05; *p* < 0.01; *p* < 0.001, where the lower the *p*-value, the more significant difference between two means.

All of the demonstrated differences are at maximum on the significance level *p* < 0.05 what indicates no great impact of any corrosive factor on the material after 1000 h. For samples in soil (III) and frozen at −16 °C in both water (IX) and air (X) no statistically significant difference was found, thus those cases were excluded from further durability analysis. Statistically noticeable changes appeared for weathered samples—*E*’ increase and *W*_M_ decrease were observed (Figure 6b,e). Although at the adopted level of significance no difference in elongation was found, it can be seen in the graph in Figure 4d that weathered samples have a smaller ability to strain increase, which seems to be on the verge of the statistical range. Such a result indicates increased stiffness of the material after 1000 h of natural weathering. The mechanical changes in the weathered samples seem to become stiffer, which indicates further cross-linking due to UV radiation. Such effect was described in the literature—FTIR studies showed that residual isocyanate groups can undergo a chemical reaction, while exposed to UV radiation. [25]. Unfortunately, after the initial increase of stiffness and the number of cross-links, polyurethanes start to degrade [25,42]. It is also possible that UV degradation makes the thin film at the polyurethane surface, which protects the inner material volume against further degradation, as was mentioned in [33].

The influence of alkalis and aqueous environments is not obvious. *E*_0_ has increased for alkali at 60 °C (VI), but *E*’ has decreased for alkali at 20 °C (V), (Figure 6a,b). Moreover, alkali at 20 °C (V) showed lower σ_M_ when there was no such observation for the higher temperature (Figure 6c). *E*_0_ decrease was also observed for distilled water (IV) and seawater (VII). In addition, seawater showed significantly lower *W*_M_ (Figure 6e), and tap water lower σ_M_ (Figure 6c). It is probable that the observed small changes in the mechanical properties are caused by leaching of mineral fillers, what generated precipitation in distilled water (IV) and alkalis at 20 °C and 60 °C, (V) and (VI) respectively. This may explain the differences in the influence of aqueous environments, as they had different ion types and concentrations.

### 3.2. Thermal Analysis

**Figure 7:**
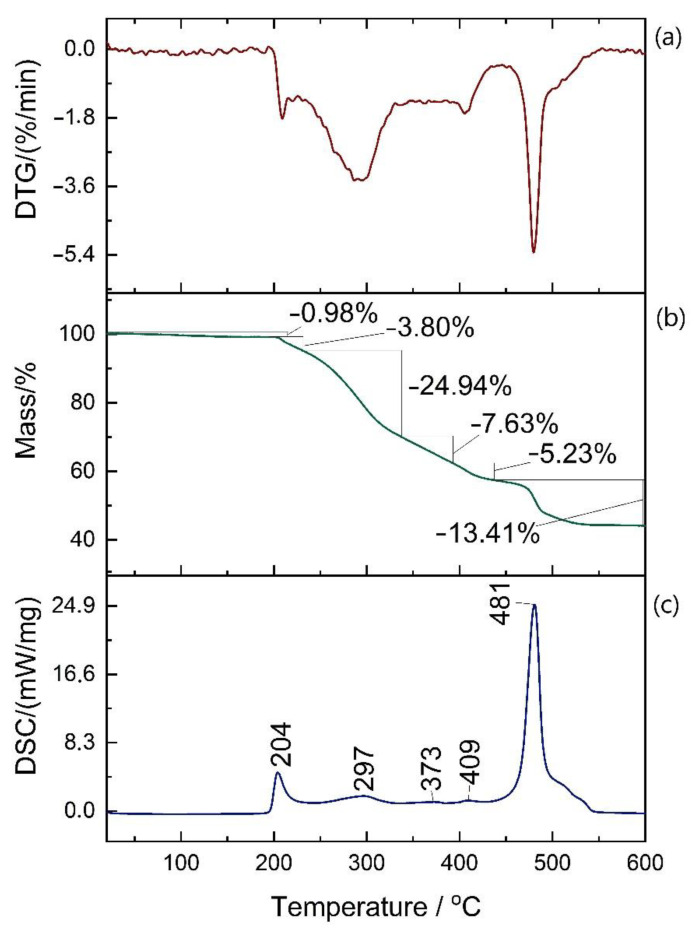
Result of DTG (**a**), TGA (**b**), and DSC (**c**) for sample 1 of the reference (I), investigated after tensile test.

Figure 7 shows DTG, TGA, and DSC curves of the same sample from reference (I) (sample 1/6). According to the DSC result, no thermal effect was observed in any sample below 200 °C. In this temperature range, only a very small mass loss was observed (0.98% here, 0.89–1.71% other reference samples) that is probably due to absorbed water and other volatile substances evaporation. By the time temperature in the chamber reached 600 °C, the samples’ mass was reduced to 42–44% of the initial value. The loss occurred mainly in five stages, each with a corresponding DSC exothermic peak—the range of peaks correlated with the range of mass losses are shown in Table 1. This indicates the segmented structure of the polymer and staged thermal degradation with every stage related to different chemical group disintegration. First to decompose should be flexible segments followed by stiff ones, from which the latter effect should come from (at approx. 480 °C). 

The investigated material is thermally stable up to 200 °C until the first exothermic effect is observed. 

**Figure 8:**
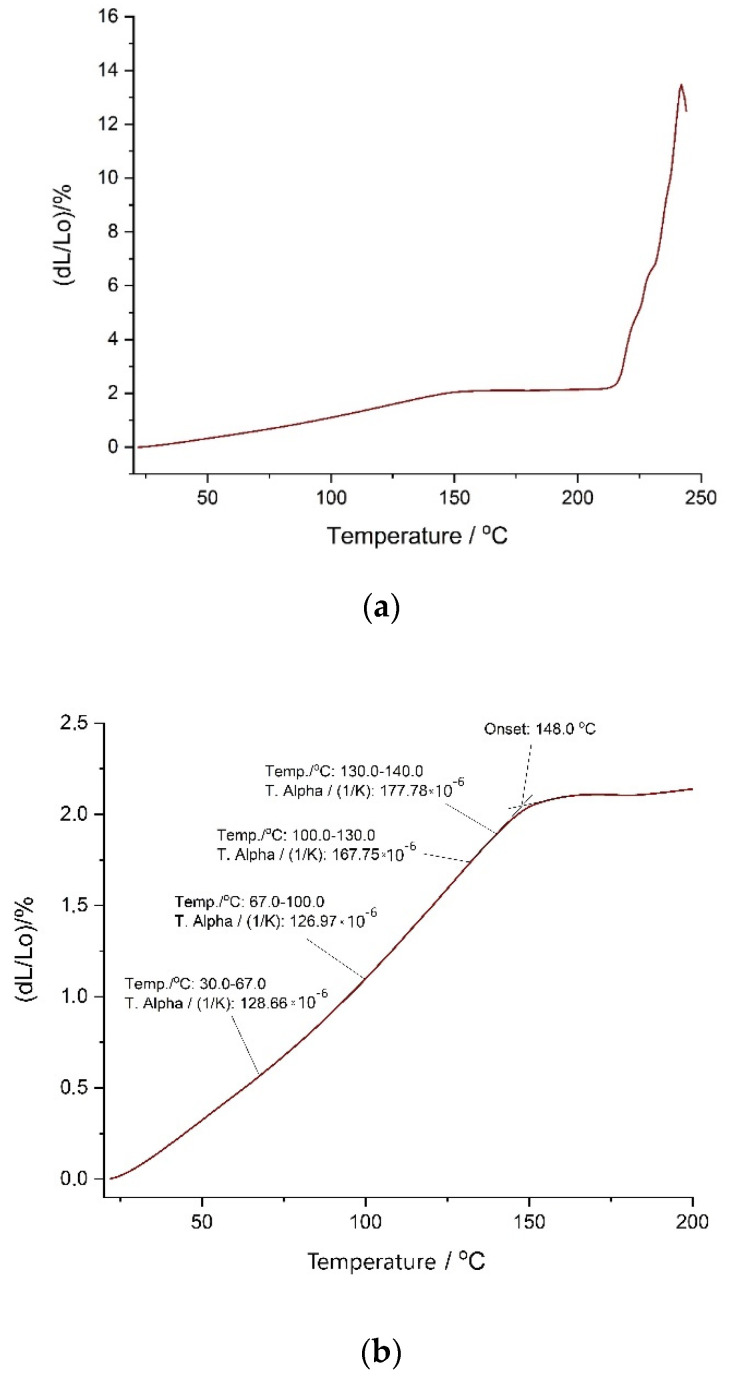

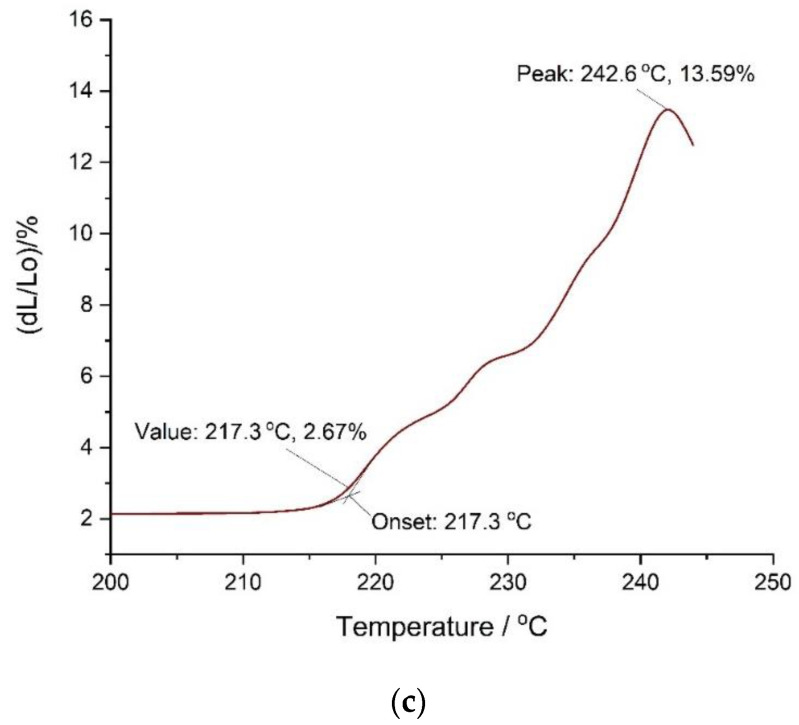
Dilatometric curve of sample 2 of reference (I). (**a**) whole measurement, (**b**) close-up on the effect in 140–150 °C, (**c**) close-up on the effect above 210 °C.

Figure 8 shows the dilatometric curve of sample 2 of reference (I) material. The experiment demonstrates a gradually increasing thermal expansion coefficient of the PS-polyurethane to the temperature around 140–150°C (onset for samples 1, 2, 3: 142.3, 148.4, 148.0, respectively). The value of the thermal expansion coefficient α for this material is about 0.00015 [1/°C] (Figure 8), which corresponds very well to the value α = 1.56e04 [1/°C] determined for Sika PS polyurethane using another testing method [37]. In the next temperature range up to 210°C, the curve flattens out, and subsequently measured thermal expansion is much lower—see close-up in Figure 8b. The material’s elongation is about 2% to the temperature of 210 °C. Then, at a temperature around 210–220 °C (onset for samples 1, 2, 3: 210.3, 217.3, 210.1, respectively), rapid expansion begins, as the material starts swelling until the curve reaches the peak (for samples 1, 2, 3: 229.0, 242.6, 236.8, respectively)—see close-up in Figure 8c. Then, the material becomes too soft so that the apparatus moving rod dips into it and measurement is no longer possible. 

Polymer swelling followed by rapid softening (Figure 8c) is clearly correlated with the first exothermic effect observed during DSC measurement—it is the consequence of first stage degradation. More complicated is however the other effect (close-up in Figure 8b). The rapid change in the behavior suggests some kind of transition within the structure with no corresponding DSC peak (Figure 7a) observed. To explain the origin of this effect, further research is required, but it does not indicate the failure of mechanical properties. 

### 3.3. SEM and EDS Analysis

**Figure 9:**
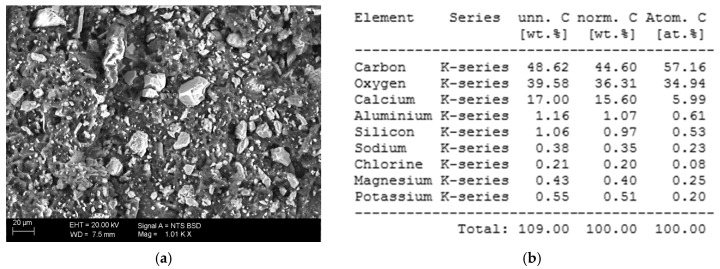

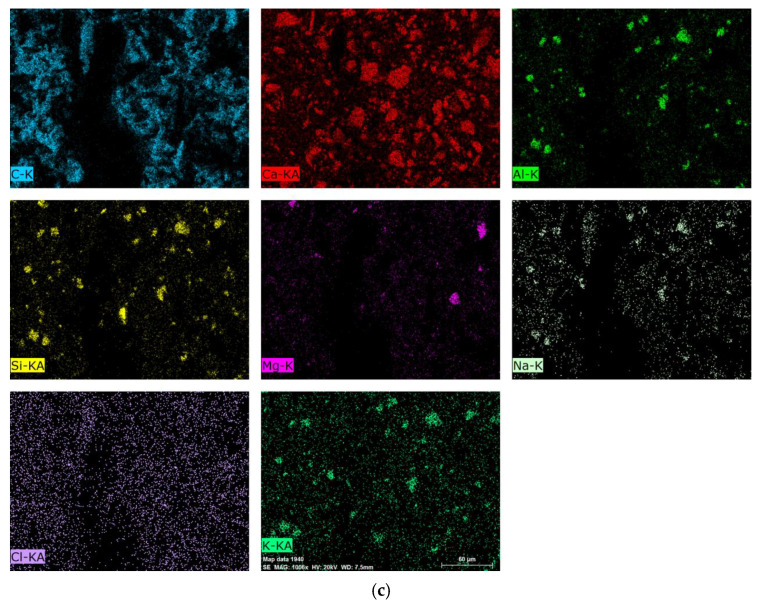
(**a**) SEM image of the unaged polymer (I), magnification 1000×, (**b**) analysis of the EDS elemental composition of the surface, (**c**) mapping of the elements present in the sample.

The figures below present selected results of microscopic observations along with the EDS analysis of the unaged polymer (I) (Figure 9). Carbon visible on EDS mapping is the main element of polymer chains. Aluminum and silicon are accumulated together with the presence of sodium and potassium, which suggests that they are mineral fillers in the form of aluminosilicates. Chlorine also seems to come as sodium and potassium chloride. A relatively big amount of calcium indicates its role as a filler too, in the most probable form of calcium carbonate, that could be mixed with co-appearing magnesium carbonate. 

**Figure 10:**
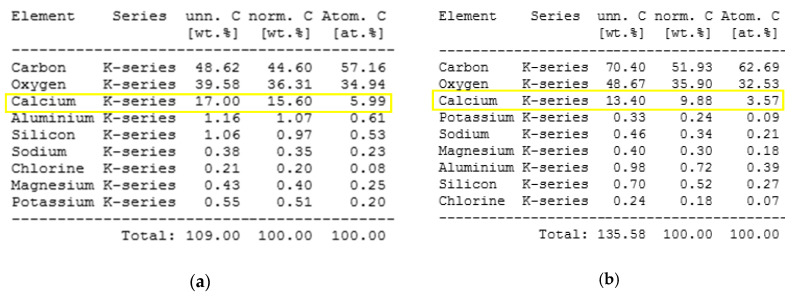

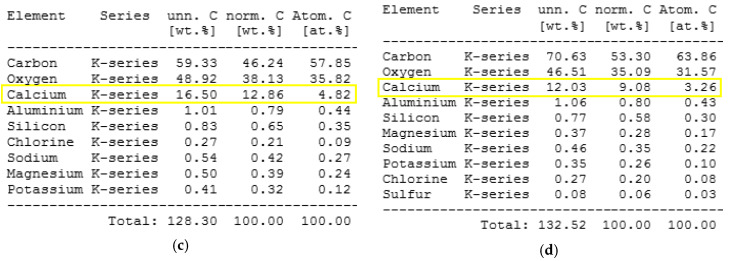
EDS surface analysis of (**a**) reference (I), (**b**) distilled water (IV), (**c**) alkalis at 60 (VI), (**d**) sea water (VII). The changes of calcium content on the surface have been marked with yellow boxes—decreasing values suggest elution of mineral fillers.

EDS analysis of the polymer aged in distilled water at 60 °C (IV), the polymer aged in Ca(OH)_2_ solution at 60 °C (VI), and the polymer aged in seawater at 20 °C (VII) are shown in Figure 10. The most significant changes in the samples’ surface are according to the decreasing amount of calcium due to immersion in aqueous environments. Mineral filler can undergo dissolution to a certain degree, especially in distilled water. This effect is least seeable in the Ca(OH)_2_ solution, as the presence of Ca^2+^ cations inhibits this process. Also, the appearance of sulfur in the case of seawater suggests reactive bonding sulfate anions occurring in sea water on the surface with the creation of insoluble gypsum.

**Figure 11:**
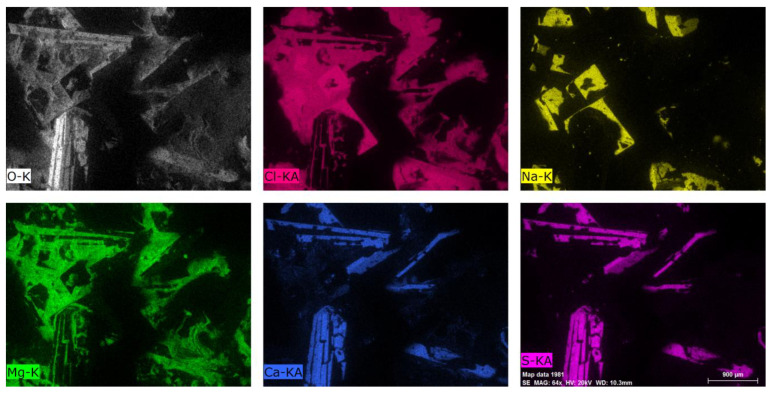
EDS surface analysis of crystals obtained in distilled water (IV).

In the case of polymer samples aged in distilled water at 60 °C, the crystallized product on their surface (after partial evaporation of water) was also investigated in the same technique. The results of the microscopic examination of the formed crystals are shown in Figure 11. Eluted salts recrystallized in the case of distilled water as immersion fluid (Figure 5c). EDS analysis showed that the crystals consist of the same elements as were found on the polymer surface. As seen in Figure 11, they are gypsum (calcium sulfate), sodium chloride, magnesium chloride, and magnesium oxide. This also indicates that solid residue in alkalis at 20 °C (V) and 60 °C (VI) (Figure 5a,b) are at most the mixture of gypsum and recrystallized calcium hydroxide due to increased Ca^2+^ concentration. Different forms of precipitates probably have their origin in nucleation kinetics being influenced by temperature.

## 4. Conclusions

Materials for civil engineering purposes must show the durability of their mechanical properties. We successfully checked the influence of chosen corrosive environments on PS-polyurethane by tensile test, and in general, no serious changes in this field were observed.

- Specimens subjected to soil (III), frozen water (IX), and cold air (X) showed no significant changes in any of the analyzed parameters. These three corrosive factors are considered to be safe for investigated material.
- Specimens subjected to five various aqueous environments showed mild but different changes. Applied time (1000 h) is however relatively short for such an experiment, so longer exposition of the samples on corrosive factors should be tested. We suppose that the changes come from elution and chemical reactions of mineral fillers which are in the form of very fine particles in the size of several dozen µm. They may affect the behavior of the material similarly as in composites. In such a case, no structural changes within the polymer itself would be present. However, this should be investigated in further steps via FTIR (Fourier transformed infrared) spectroscopy to check whether any chemical changes in polyurethane chains are present.
- Specimens subjected to natural weathering increased slightly their stiffness. This is however a reported behavior of polyurethanes at the beginning of photodegradation. A relatively short time and mild natural UV-exposure suggest that the investigated polymer should behave similarly and the observations are signs of initial degradation. Although this happens, the process takes place on the surface and may in fact protect the inner material.

Thermal stability of PS-polyurethane in a lower temperature range up to 100 °C had been proven before [36,37]. The concern was however raised about the conditions of a fire. Performed DSC-TGA tests and dilatometric studies showed:

- Neither thermal effect nor significant mass loss in elevated temperatures up to 200 °C.
- Right above 200 °C swelling of the polymer, followed by rapid loss of mechanical properties occurs
- There is an effect observed between 140–150 °C on the dilatometric curve that needs further studies to determine its origin, but it does not seem to worsen mechanical properties of the polymer
- Where the application material will be covered within the walls, this should increase its temperature gradually without rapid failure, enabling people to escape from a burning building. To define the exact time which PS-polyurethane can withstand under fire conditions, more specific research is required, but preliminary results are very promising.

In conclusion, PS-type polyurethane seems to be able to withstand environmental conditions in possible places of application. Neither alkalis from mortars nor humidity nor temperature changes up to 200 °C should affect such technological solution, what confirms its safe usage in different civil engineering applications. These were, however, preliminary studies which showed some interesting outcomes and suggest further research, that should be provided to confirm presented conclusions and to describe PS-polyurethane behavior more in detail. 

## Figures and Tables

**Table 1:** Percentage mass losses intervals in 5 degradation stages with corresponding DSC peak intervals.

STAGE	1	2	3	4	5
MASS LOSS [%]	3.20–4.29	20.55–26.12	6.75–13.48	3.68–6.22	11.79–13.74
PEAK TEMP [°C]	204–207	294–306	338–378	404–409	476–481

## References

[1] Earthquake Disaster Relief. https://www.unicefusa.org/mission/emergencies/earthquakes.

[2] Viskovic A., Zuccarino L., Kwiecień A., Zając B., Gams M. (2017). Quick seismic protection of weak masonry infilling in filled framed structures using flexible joints. Key Eng. Mater..

[3] Kwiecień A., Krajewski P., Hojdys Ł., Tekieli M., Słoński M. (2018). Flexible Adhesive in Composite-to-Brick Strengthening—Experimental and Numerical Study. Polymers.

[4] Derkowski W., Kwiecień A., Zajac B. (2013). CFRP strengthening of bent RC elements using stiff and flexible adhesives. Tech. Trans..

[5] Kwiecień A. (2013). Highly deformable polymers for repair and strengthening of cracked masonry structures. Gstf Int. J. Eng. Technol..

[6] Kwiecień A. Polymer flexible joints—An innovative repair system protecting cracked masonries against stress concentrations. Proceedings of the International Conference on Protection of Historical Buildings.

[7] Kwiecień A. (2010). New repair method of cracked concrete airfield surfaces using of polymer joints. Proceedings of the ICPIC 2010.

[8] Akyildiz A.T., Kwiecień A., Zając B., Triller P., Bohinc U., Rousakis T., Viskovic A. (2020). Preliminary in-Plane Shear Test of Infills Protected by PUFJ Interfaces.

[9] Rousakis T., Kwiecień A., Zając B., Hojdys Ł., Krajewski P., Tekieli M., Akyildiz T. Flexible joints between RC frames and masonry infill for improved seismic performance–shake table tests. Proceedings of the 17th International Brick and Block Masonry Conference (17th IB2MAC 2020).

[10] Lasowicz N., Kwiecień A., Jankowski R. (2015). Experimental study on the effectiveness of polymer damper in damage reduction of temporary steel grandstand. J. Phys. Conf. Ser..

[11] Lasowicz N., Kwiecień A., Jankowski R. (2020). Experimental Study on the Effectiveness of Polyurethane Flexible Adhesive in Reduction of Structural Vibrations. Polymers.

[12] Kwiecień A. (2014). Shear bond of composites-to-brick applied with highly deformable, in relation to resin epoxy, interface materials. Mater. Struct..

[13] De Santis S., Ceroni F., de Felice G., Fagone M., Ghiassi B., Kwiecień A., Lignola G.P., Morganti M., Santandrea M., Valluzzi M.R. (2017). Round Robin Test on tensile and bond behaviour of Steel Reinforced Grout systems. Compos. Part. B Eng..

[14] Cruz J.R., Seręga S., Sena-Cruz J., Pereira E., Kwiecień A., Zając B. (2020). Flexural behaviour of NSM CFRP laminate strip systems in concrete using stiff and flexible adhesives. Compos. Part. B Eng..

[15] Ghiassi B., Xavier J., Oliveira D.V., Kwiecien A., Lourenço P.B., Zajac B. (2015). Evaluation of the bond performance in FRP–brick components re-bonded after initial delamination. Compos. Struct..

[16] Kwiecień A., de Felice G., Oliveira D.V., Zając B., Bellini A., De Santis S., Ghiassi B., Lignola G.P., Lourenço P.B., Mazzotti C. (2016). Repair of composite-to-masonry bond using flexible matrix. Mater. Struct..

[17] Sonnenschein M.F. (2015). Introduction to Polyurethane Chemistry. Polyurethanes: Science, Technology, Markets, and Trends.

[18] Hepburn C. (2012). Polyurethane Elastomers.

[19] Singh B., Sharma N. (2008). Mechanistic implications of plastic degradation. Polym. Degrad. Stab..

[20] Xie F., Tianlong Z., Bryant P., Kurusingal V., Colwell J.M., Laycock B. (2019). Degradation and stabilization of polyurethane elastomers. Prog. Polym. Sci..

[21] Gadea J., Rodriguez A., Campos P.L., Garabito J., Calderón V. (2010). Lightweight mortar made with recycled polyurethane foam. Cem. Concr. Compos..

[22] Junco C., Gadea J., Rodríguez A., Gutiérrez-González S., Calderón V. (2012). Durability of lightweight masonry mortars made with white recycled polyurethane foam. Cem. Concr. Compos..

[23] Kim Y.D., Kim S.C. (1998). Effect of chemical structure on the biodegradation of polyurethanes under composting conditions. Polym. Degrad. Stab..

[24] Rutkowska M., Krasowska K., Heimowska A., Steinka I., Janik H. (2002). Degradation of polyurethanes in sea water. Polym. Degrad. Stab..

[25] Yang X.F., Vang C., Tallman D.E., Bierwagen G.P., Croll S.G., Rohlik S. (2001). Weathering degradation of a polyurethane coating. Polym. Degrad. Stab..

[26] Hawkins W.L. (1984). Polymer Degradation and Stabilization.

[27] Zhang Y., Xia Z., Huang H., Chen H. (2009). Thermal degradation of polyurethane based on IPDI. J. Anal. Appl. Pyrolysis.

[28] Sarkar S., Adhikari B. (2001). Thermal stability of lignin–hydroxy-terminated polybutadiene copolyurethanes. Polym. Degrad. Stab..

[29] Chen H., Lu H., Zhou Y., Zheng M., Ke C., Zeng D. (2012). Study on thermal properties of polyurethane nanocomposites based on organo-sepiolite. Polym. Degrad. Stab..

[30] Król P., Pilch-Pitera B. (2007). Phase structure and thermal stability of crosslinked polyurethane elastomers based on well-defined prepolymers. J. Appl. Polym. Sci..

[31] Howard G.T. (2002). Biodegradation of polyurethane: A review. Int. Biodeterior. Biodegrad..

[32] Nakajima-Kambe T., Shigeno-Akutsu Y., Nomura N., Onuma F., Nakahara T. (1999). Microbial degradation of polyurethane, polyester polyurethanes and polyether polyurethanes. Appl. Microbiol. Biotechnol..

[33] Kozak A., Kwiecie A. Accelerated weathering tests of polyurethane mass for flexible joints to repair concrete and masonry structural elements. Proceedings of the 7th International Conference AMCM 2011.

[34] Tedeschi C., Kwiecień A., Valluzzi M.R., Zając B., Garbin E., Binda L. (2014). Effect of thermal ageing and salt decay on bond between FRP and masonry. Mater. Struct..

[35] De Santis S., Stryszewska T., Bandini S., de Felice G., Hojdys Ł., Krajewski P., Kwiecień A., Roscini F., Zając B. (2018). Durability of steel reinforced polyurethane-to-substrate bond. Compos. Part. B Eng..

[36] Zając B., Kwiecień A., Gams M., Tatara T. Strengthening of masonry and concrete structures working in elevated temperatures and mining tremors area. Proceedings of the 5th International Scientific Conference on Civil Engineering-Infrastructure-Mining.

[37] Zając B., Kwiecień A., Aguilar R., Torrealva D., Moreira S., Pando M.A., Ramos L.F. (2019). Thermal Compatibility of Rigid and Flexible Adhesives to Substrates of Historical Structures. Structural Analysis of Historical Constructions.

[38] (2019). ISO 527-1:2019 Plastics—Determination of Tensile Properties—Part. 1: General Principles.

[39] Haynes W., Dubitzky W., Wolkenhauer O., Cho K.-H., Yokota H. (2013). Tukey’s Test. Encyclopedia of Systems Biology.

[40] Kwiecień A., Gams M., Zając B. Numerical modelling of flexible polymers as the adhesive for FRPs. Proceedings of the FRPRCS-12 & 5th Asia-Pacific Conference on Fiber Reinforced Polymers in Structures (APFIS-2015).

[41] Kwiecień A. (2015). Constitutive equations modelling of hyperelastic polymers in flexible joints [in polish]. Współczesna Mechanika Konstrukcji w Projektowaniu Inżynierskim.

[42] Cui H., Hanus R., Kessler M.R. (2013). Degradation of ROMP-based bio-renewable polymers by UV radiation. Polym. Degrad. Stab..

